# iOmicsPASS: network-based integration of multiomics data for predictive subnetwork discovery

**DOI:** 10.1038/s41540-019-0099-y

**Published:** 2019-07-09

**Authors:** Hiromi W. L. Koh, Damian Fermin, Christine Vogel, Kwok Pui Choi, Rob M. Ewing, Hyungwon Choi

**Affiliations:** 1 Department of Medicine, Yong Loo Lin School of Medicine, National University of Singapore, Singapore, Singapore; 2 Saw Swee Hock School of Public Health, National University of Singapore, Singapore, Singapore; 3 University of Michigan Medical School, Ann Arbor, MI USA; 4 Center for Genomics and Systems Biology, Department of Biology, New York University, New York, NY 10003 USA; 5 Department of Statistics and Applied Probability, National University of Singapore, Singapore, Singapore; 6 School of Biological Sciences, University of Southampton, Southampton, UK; 7 Institute of Molecular and Cell Biology, Agency for Science, Technology and Research, Singapore, Singapore

**Keywords:** Computational biology and bioinformatics, Systems biology

## Abstract

Computational tools for multiomics data integration have usually been designed for unsupervised detection of multiomics features explaining large phenotypic variations. To achieve this, some approaches extract latent signals in heterogeneous data sets from a joint statistical error model, while others use biological networks to propagate differential expression signals and find consensus signatures. However, few approaches directly consider molecular interaction as a data feature, the essential linker between different omics data sets. The increasing availability of genome-scale interactome data connecting different molecular levels motivates a new class of methods to extract interactive signals from multiomics data. Here we developed iOmicsPASS, a tool to search for predictive subnetworks consisting of molecular interactions within and between related omics data types in a supervised analysis setting. Based on user-provided network data and relevant omics data sets, iOmicsPASS computes a score for each molecular interaction, and applies a modified nearest shrunken centroid algorithm to the scores to select densely connected subnetworks that can accurately predict each phenotypic group. iOmicsPASS detects a sparse set of predictive molecular interactions without loss of prediction accuracy compared to alternative methods, and the selected network signature immediately provides mechanistic interpretation of the multiomics profile representing each sample group. Extensive simulation studies demonstrate clear benefit of interaction-level modeling. iOmicsPASS analysis of TCGA/CPTAC breast cancer data also highlights new transcriptional regulatory network underlying the basal-like subtype as positive protein markers, a result not seen through analysis of individual omics data.

## Introduction

Today’s systems biology research frequently employs two or more omics platforms such as massively parallel sequencing and mass spectrometry to identify systemic patterns in biological signals from different types of molecules. It is a complex task to synthesize findings from multiple sets of heterogeneous data and to tease out easily interpretable feature sets explaining phenotypic variation, often from a limited number of observations. Therefore, efficient computational frameworks that can integrate data sets with proper biological prior are of paramount importance.

Numerous data analysis software packages are already available for multiomics data integration in different contexts.^1^ Sample clustering via multiomics data integration is a popular application, as unsupervised analysis creates abundant opportunities to extract different types of signals such as latent factors (LFs), without being confined to prespecified sample groups that may or may not be a major source of variation in the data. For example, iCluster^2^ and its recent extension iClusterPlus^3^ are successful model-based solutions that extract shared LFs with varying contributions from individual omics data sets and cluster subjects in the space of identified factors. Patient-specific data fusion also offers a highly flexible approach to model each individual’s multiomics profile as an outcome of subject-specific feature sets, while providing stratification of subjects into clusters with automatic selection of an optimal number of clusters, all achieved by Bayesian nonparametric inference.^4^ A more recent approach called Multi-Omics Factor Analysis (MOFA) provides a computationally efficient group factor analysis method equipped with mean field approximation-based Bayesian inference to account for various types of quantitation (i.e. intensities, counts, and binary status), with the ability to tease out factors that are shared across different omics data and those that are unique to each data source.^5^

While model-based approaches have proven to be efficient for sample-level analysis, not all prioritized data features, such as the loading scores of individual molecules in LFs, immediately align well with known mechanistic links between molecules within each omics type (e.g. physical binding of two proteins), or between different omics types (e.g. regulation of a transcription factor (TF) proteins and mRNAs of its target genes.) Network-based approaches address this lack of biological interpretability by incorporating experimentally acquired genome-scale biological network data into the analysis. Instead of completely relying on mathematical deconvolutions to identify latent structures, these approaches borrow prior information from experimentally tested or predicted interactions to overlay heterogeneous multiomics data and overcome the inherent noise in data sets of a small sample size.

The latter class of methods is best exemplified by PARADIGM,^6^ an unsupervised analysis method to infer patient-specific pathway activation and deactivation status by formulating the underlying probability model of multiomics data as factor graphs.^6,7^ LemonTree finds coexpressed gene clusters and reconstructs regulatory programs involving other upstream omics data as network modules.^7^ Other methods have also taken system-level data summarization approaches such as network propagation algorithms to merge signals from mutations and gene expression data, detecting gene signatures that would otherwise be missed in association analysis for disease phenotypes if the individual data sets had been analyzed in isolation.^8^

Despite these developments, few software implementations offer a data integration approach that combines multiomics measurements over networks in a way that (i) prioritized molecular features immediately reveal functional relations between themselves and (ii) the molecular levels of the features are directly relevant to the given type of networks. For example, many network-based integrative analyses have merged mutation data and/or transcriptomic data over protein–protein interaction (PPI) data.^9^ However, given the relatively modest correlation among DNA copy number, mRNA, and protein expression noted over a number of studies,^10,11^ it is more desirable to integrate protein expression data of two physically binding proteins rather than at the DNA or mRNA level data. Likewise, if a TF is known to regulate expression of a target gene, then the relevant data types are protein abundance of the TF and mRNA expression of the target gene.

To fill this gap, we developed a network-based method iOmicsPASS to integrate multiomics profiles over genome-scale biological networks and identify sparse subnetworks predictive of prespecified phenotypes. iOmicsPASS performs two main tasks: (i) integrate quantitative multiomics data consisting of DNA copy number (optional), transcriptomics and proteomics data by computing interaction scores for a given network and (ii) discover a set of molecular interactions whose joint expression patterns predict phenotypic groups the best, i.e., predictive subnetworks.

We first show that iOmicsPASS accurately identifies key interactions underlying phenotype-predictive signals using simulation studies, with high sensitivity under varying network coverage. In particular, we show that our adaptive centroid calculation and group-specific shrinkage operator yield locally connected predictive subnetworks with improved predictive performance over other modes of predictive feature selection, especially against the obvious alternative of applying machine learning algorithms on the concatenated data.^12^ We next illustrate the utility of iOmicsPASS through the analysis of The Cancer Genome Atlas (TCGA) breast cancer (BRCA) data, where we integrated multiple omics profiles for mRNA expression and protein abundance, with and without the normalization of the mRNA data by the DNA copy number variation. Not only does iOmicsPASS recapitulate a network of hormone receptors and transcription regulators defining BRCA subtypes, it also expands the subtype-specific subnetworks to additional markers that have literature evidence of interactive or regulatory mechanisms relevant for subtype characterization. The scoring adjustment we introduced especially highlighted (TF) regulatory networks positively regulated in the basal-like subtypes, where few positive markers have been delineated.

## Results

### Overview of iOmicsPASS workflow

iOmicsPASS takes quantitative multiomics data and biological networks as input, and it calculates interaction scores for all molecular interactions in the network. The interaction scores are subsequently used for predictive subnetwork discovery. Some biological networks are nondirectional in nature (e.g., physical or genetic interactions), while others are inherently directional (e.g., TF regulatory networks). iOmicsPASS treats all network data as undirected graphs in the derivation of interaction scores and avoids modeling the full conditional probability structure for the directional networks. This design was chosen considering the fact that most input data sets used for analysis are cross-sectional expression data sets and thus it is more sensible to focus on coexpression of two interacting molecules, rather than directly incorporating the fact that one molecule ‘regulates’’ the expression of the other. This choice renders the algorithmic design generic to the integration of various types of multiomics data (directional and nondirectional), including pairs of template and product molecules (DNA and mRNA), physically binding partners (proteins), or transcription/translation regulatory element and its target. Our current implementation focuses on the integration of mRNA and protein data over TF regulatory networks and PPI networks (with or without DNA copy number variation data.)

**Fig. 1:**
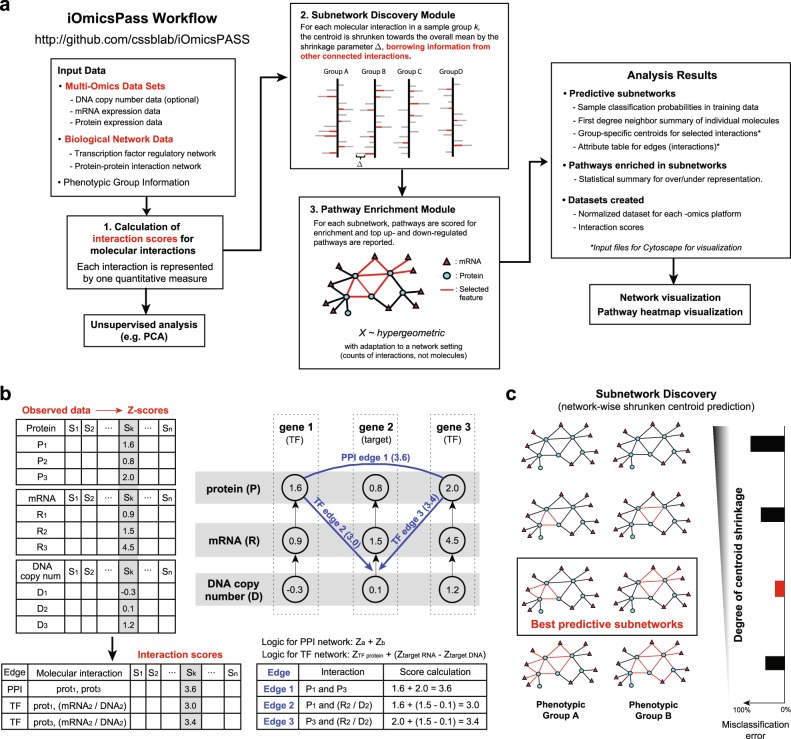
**a** iOmicsPASS workflow. iOmicsPASS takes multiomics data, biological network data, and sample meta information as input. The omics data sets are integrated via interaction scores for all interactions in the network. Subnetwork discovery module discovers the subnetwork signatures distinguishing phenotypic groups, and pathway enrichment module reports associated biological processes. The software also produces a set of text files containing the details of the selected subnetworks and the materials for visualization of networks in the Cytoscape software. **b** Each omics data is first standardized into *Z*-scores and converted to interaction scores over the network. Two TFs (gene 1 and gene 3) and their common target gene (gene 2) are shown as an example. Interaction scores are computed for the PPI between protein 1 and protein 3 and the transcription factor regulation between the two TF proteins and mRNA molecule of their target gene. **c** The resulting interaction scores are used as an input to select the predictive edges for phenotypic groups using the modified nearest shrunken centroid algorithm

Figure 1a shows the three analysis modules in the iOmicsPASS workflow: (1) transformation of quantitative multiomics data into scores for biological interactions; (2) selection of predictive subnetworks from the composite network by a modified shrunken gene-centroid algorithm;^13^ and (3) reporting of biological pathways enriched in the subnetwork selected for each phenotypic group.

The key first step in our workflow is transforming the quantitative data of individual molecules into interaction scores (Fig. 1b). We derive a score for each edge connecting two interacting molecules from their respective *Z*-scores, assuming that simultaneously high or low expression indicates high or low chance of the interaction, respectively (see “Methods”). For example, protein abundance of a TF gene and mRNA expression of its target gene can be integrated to infer the activation potential of a TF regulatory network. If both the protein abundance of the TF gene and the mRNA expression of the target genes have high *Z*-scores in a sample (leading to high interaction scores), then we assume that elevated abundance of the TF has contributed to the upregulation of mRNAs of the target genes in that sample. Likewise, if two physically binding proteins have simultaneously high *Z*-scores, it indicates that there is an increased chance of physical binding between the two, although additional determinants of actual binding such as post-translational modifications will have to adjudicate this conclusion.

Next, the resulting interaction scores are used as input to the subnetwork discovery module. The module embodies a modified version of the nearest shrunken centroid (NSC) classification algorithm, a simple yet powerful method originally developed for gene expression microarray data.^13^ The NSC algorithm treats individual features as independent Gaussian random variables searches and selects a sparse set of predictive features (Fig. 1c). The method is our preferred choice for further adaptation to build a network-oriented feature selection method since, unlike regression-based methods, it does not require choice of a reference group and it is thus naturally amenable for multiclass classification problems.

In iOmicsPASS, we introduced a group-specific shrinkage operator to render the network signature selection unbiased for networks of varying sizes across sample groups, and adjusted the calculation of centroids in a way that favors densely connected networks over scattered networks as predictive signatures. Based on the interaction scores, our modified NSC algorithm searches for sparse and well-connected subnetworks that predict phenotypes with the smallest misclassification error (cross validation). See “Methods” for details.

The analysis pipeline in iOmicsPASS reports several key results in separate text files: (i) a file containing interaction scores, which can be used for further analysis such as principal component analysis (PCA); (ii) predictive subnetworks for phenotypic groups with group-specific centroids; (iii) data files to visualize the predictive subnetworks in the Cytoscape environment;^14^ and (iv) a table of pathways enriched in the predictive subnetworks. We illustrate these functionalities below.

### Simulations: iOmicsPASS recovers dense predictive networks

We first conducted comprehensive simulation studies to evaluate the prediction performance of iOmicsPASS in comparison with other approaches: (i) NSC algorithm applied to concatenated multiomics data; (ii) Support Vector Machine (SVM), a widely used kernel learning algorithm in machine learning; and (iii) iOmicsPASS without the modified centroid shrinkage that favors densely connected predictive subnetworks. Here our primary goal is to show that, when the underlying data generation scheme is completely or partially captured by the given network, iOmicsPASS’s feature selection method not only achieves comparable prediction performance to the kernel learning methods but also produces a sparse set of easily interpretable biological interactions, a property not usually offered by prediction tools of high complexity.

Supplementary Fig. 1 shows the overall simulation design and parameters used to generate the quantitative data sets. Protein expression data for 1000 TF genes and mRNA expression data for 5000 target genes were simulated 100 times, in which true TF activation signals were planted for 10% of the TF proteins and their direct mRNA gene targets with probabilities proportional to the number of TFs that target them. The signal-to-noise ratio (setup A, B, and C) and the assay sensitivity parameter (*P*_AS_) were set to reflect the properties of the TCGA BRCA data set, and the TF network was also derived from the network data assembled for human data (see “Methods”) in order to emulate the topology of TF regulatory networks in human cells. Here, 48,682 possible TF-target regulatory interactions were constructed between the molecules, and of those, 6742 (13.8%) were regarded as edges with true signals.

**Fig. 2:**
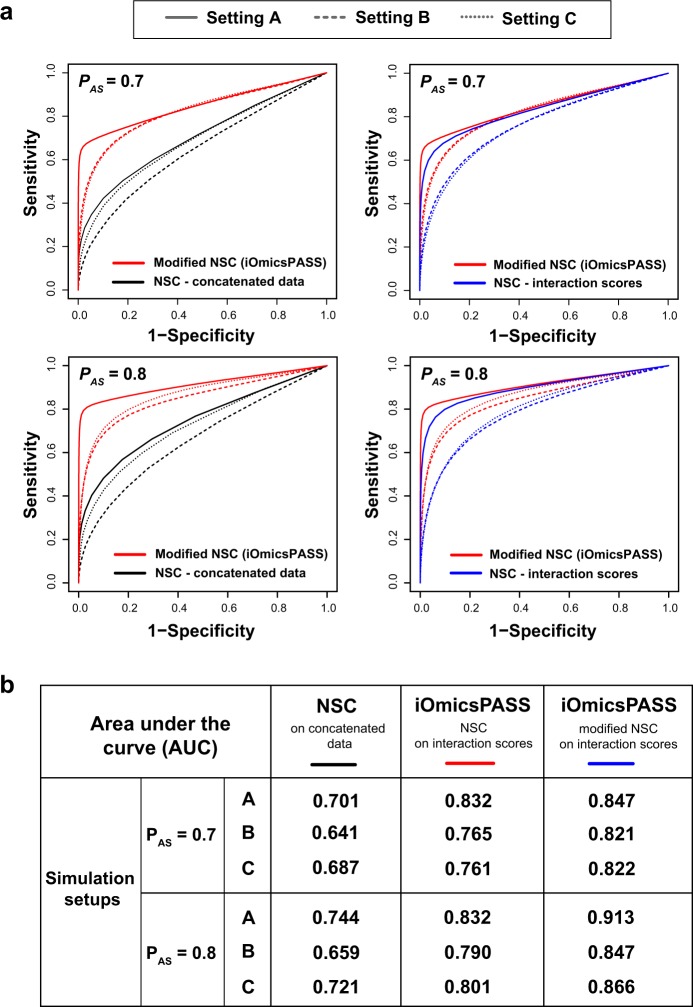
**a** Simulation results using the NSC algorithm applied to the concatenated data (black lines), the NSC algorithm to the interaction scores (blue lines), and the modified NSC algorithm to the interaction scores in iOmicsPASS (red lines). Six different parameters determining the levels of signal and noise were used to simulate data based on a biological network sampled from a real TF and PPI network. **b** Area under the curve (AUC) of three approaches, each represented by one colored line in **a**, using three simulation setups at assay sensitivity values of 0.7 and 0.8

We first compared iOmicsPASS with the original NSC algorithm applied to the concatenated dual-omics data sets. Figure 2 shows that the receiver operating characteristic curves of iOmicsPASS (red lines) are consistently superior to those of the NSC algorithm (black lines) with concatenated data across the combinations of signal-to-noise ratio and assay sensitivity. When assay sensitivity of proteomics was set at 0.7, the AUC improved more than 20% in setting A (AUC of the original NSC = 0.701; AUC of iOmicsPASS = 0.847), where the planted signal-to-noise ratio was the strongest. The comparative performance remained similar in settings B and C: iOmicsPASS with AUC values of 0.821 and 0.822, respectively, the NSC-based predictions had the smallest AUC (AUC = 0.641) in setting B with weaker signals. Overall, the simulation studies suggest that there is a clear benefit in selecting predictive features from a list of interactions based on interaction scores, rather than from measurements of individual molecules.

Furthermore, we evaluated the impact of the score adjustment in the NSC algorithm to secure locally dense subnetwork signatures in iOmicsPASS. Classification without this adjustment led to poorer performance (blue lines, Fig. 2) than that of NSC with the modified scoring algorithm (red lines). The effect of this modification was more visible in simulation setups with greater noise (simulation settings B and C compared to A): the difference in AUC was 0.061 in setting C, the largest among all three settings. Overall, the results of the simulation studies suggest that the enforcement of network modularity in the predictive signature can improve prediction accuracy considerably.

In addition, we also compared the cross-validated misclassification error rates of iOmicsPASS to that of SVM applied to the same hundred simulated data sets (concatenated data) in all three simulation settings using assay sensitivity of 0.7. We tuned the SVM classifier for optimal kernel (gamma) and cost parameters using the first set of data and applied the same parameters across all the rest of the data as this part of the implementation took long computation time. The cross-validated error rates of the SVM (radial basis function kernel, gamma 0.001, cost 0.1) were on average 57% across all three settings, suggesting that even the most flexible kernel learning algorithm was unable to find classification decision boundaries robustly when two noisy, heterogeneous data sets are concatenated. When we investigated SVM classifiers across data sets, we discovered that the number of support vectors was consistently above 90 (of 100), supporting the interpretation that the algorithm could not reasonably simplified classification boundaries from the training data.

Finally, we tested the performance of iOmicsPASS when the network information is incomplete and noisy, i.e., when the user-provided network contains spurious interactions (false positives) and lacks bona fide interactions (false negatives). To this end, we simulated noisy networks that include spurious interactions and lack a portion of true interactions (see “Supplementary Methods”). The results show that, even with a partially complete network, iOmicsPASS analyses still outperformed that of the NSC algorithm applied to the concatenated data and that of the NSC algorithm applied to the interaction scores across all three simulation setups (Supplementary Fig. 2).

### TF and PPI networks predictive of breast cancer subtypes

Next, we tested the ability of iOmicsPASS to discover predictive subnetworks for BRCA subtypes. We used the invasive ductal BRCA data of TCGA as a benchmark data set, with four intrinsic subtypes defined by the mRNA-based PAM50 signature as phenotypic groups.^15^ The objective of our analysis is twofold: (i) evaluate the ability of iOmicsPASS to correctly classify tumors to predefined mRNA-based subtypes using multiomics data and (ii) identify combined TF regulatory and PPI subnetworks predictive of each subtype.

To this end, we integrated DNA copy number, transcriptomics, and proteomics data produced by the TCGA^16^ and Clinical Proteomic Tumor Analysis Consortium (CPTAC),^17^ respectively. TCGA BRCA cohort has a total of 1098 tumor samples, and 103 of those had all three omics data available. TCGA assigned 24 samples to Basal-like, 18 to HER2E, 29 were luminal A, and 32 were luminal B subtype. In our main analysis, all three types of omics data were provided as input into the software. iOmicsPASS used DNA copy number to normalize the transcriptomic data of respective genes and mapped the transcriptomics and proteomics data to the TF and PPI networks.

Supplementary Fig. 3 shows the individual PCA plots using all features data in individual omics data, illustrating the level of heterogeneity of the three data sets in terms of the contribution to the largest variation (e.g., principal component 1). In particular, the plot for the proteomic data suggest the presence of variation unrelated to the separation of the four subtypes. This turned out to be a data quality issue for a portion of samples, and we will return to discuss this point later in the “Results” section.

Nevertheless, the supervised iOmicsPASS analysis overcame the heterogeneity of data sets, demonstrating good separation of subtypes in the integrated space. Supplementary Fig. 4a shows the PCA plot of the integrated interaction scores for the subnetworks selected by iOmicsPASS, suggesting that there exist subnetwork signatures that separate the four subtypes in the integrated data. One notable feature is that the identity of HER2E subtype is unclear in this sample projection plot, which can be largely considered as a part of luminal subtypes (with some of them later misclassified as luminal A in the supervised analysis.) The largest variation along the first principal component represents the separation between the luminal A subtype and the rest, while the second principal component separates the basal-like subtype from the rest. These observations clearly suggest that the proteomic data captured additional heterogeneity within and across the PAM50 subtypes.

Meanwhile, the supervised analysis via the predictive subnetwork module identified subtype-specific subnetworks consisting of 2880 molecular interactions including 647 proteins and 871 mRNAs (Supplementary Table 1). Although the overall cross-validated test error rate was ~30%, the misclassification errors were observed to be the highest for HER2E subtype (training error 55.6% and test error 85.0% from cross validation). This is consistent with the observation from the PCA plot, where out of the 18 HER2E tumors, eight were classified as luminal A subtype (green triangles) and two were classified as basal-like subtype (green solid circles) as shown in Supplementary Fig. 3b.

**Fig. 3:**
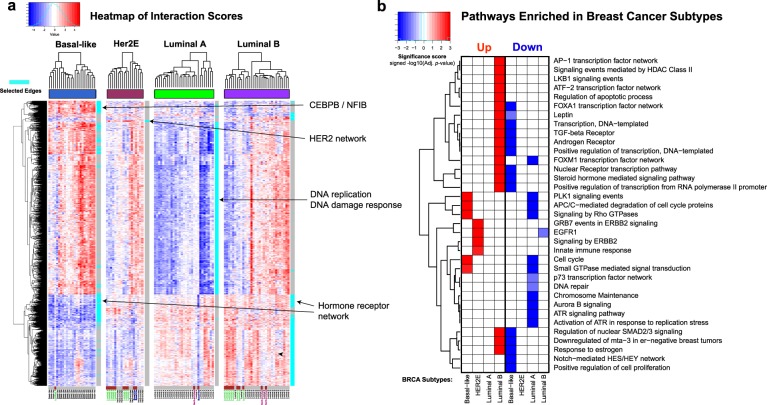
**a** Heatmap of the interaction scores for the union of all four subnetworks in the BRCA data. The cyan color bar on the right-side highlights the subtype-specific subnetworks. **b** Heatmap of statistical significance scores for the pathway enrichment in the subtype-specific subnetworks. The significance score was calculated as minus the logarithm (base 10) of Benjamini–Hochberg adjusted *p*-value. For downregulated pathways that were enriched with genes or proteins with lower interaction scores, we multiplied −1 to the significance score to make the score negative. Red and blue represent the direction of interaction scores (positive and negative, respectively)

The heatmap of interaction scores in Fig. 3a provides further insight to why the integrated scores do not completely separate HER2E subtype from the rest. Although iOmicsPASS captured simultaneous upregulation of Erb-b2 (HER2) and GRB7 protein expression in the HER2E predictive signature, this predictive subnetwork was overwhelmed by the network of luminal A-predictive TF regulations and PPIs in the DNA replication and DNA damage response network that divided HER2E tumors into two sub-groups. The heterogeneity in this subnetwork completely masks the differences in hormone receptors (ESR1, PGR, and AR) and HER2 levels, leading to classification of those tumors into luminal A subtype. This drawback can be addressed by alternative ways to compute discriminant scores and assigning subjects to classes, and we further discuss this as a future extension in the “Discussion” section.

### Visualization of group-specific subnetworks and pathways

**Fig. 4:**
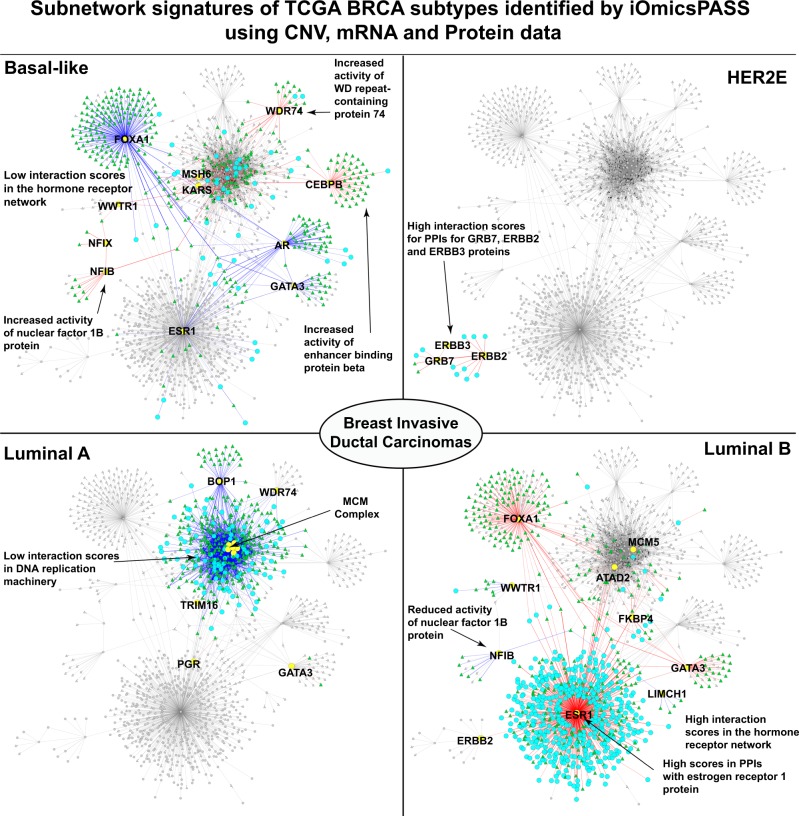
Subnetwork signatures predictive of the four intrinsic subtypes of BRCA illustrated in Cytoscape. Red and blue lines (edges) are interactions (TF regulation or PPI) with higher and lower interaction scores compared to the overall centroid, i.e. average profile in the data set. In each subtype-specific network, the proteins are indicated by cyan-colored circles and the mRNAs are green-colored triangles. Gray-colored nodes are not a part of the predictive subnetwork in a given subtype. Yellow-colored nodes indicate hub proteins of subnetworks for each breast cancer subtype

Figure 4 shows the organization of the subnetworks across the four subtypes selected by iOmicsPASS, generated by plugging its text output into Cytoscape (proteins in circles and mRNAs in triangles.) The network organization clearly demonstrates that the incorporation of proteomics data not only singled out the diagnostic markers of the hormone receptors specifically but also captured protein-level downregulation of the DNA repair machinery in luminal A subtype, the group known to have the best prognosis. In the latter, the downregulated subnetwork highlighted in blue in luminal A subtype in Fig. 4, consists of a multitude of protein complexes and (TF) regulatory networks involving DNA replication and DNA-repair pathways (see Supplementary Table 1 for detailed subnetwork information.)

One of the most interesting features in the network diagram is that several transcriptional regulators, including CEBPB, NFIB, WWTR1, and WDR74, were selected as positive, not negative, markers of the basal-like subtype. We circle back to this point later when we discuss the impact of mRNA expression data normalization by the DNA copy number. Nonetheless, the edge-level analysis clearly highlighted protein-level evidence of the transcription regulators as the unique driver of basal-like subtype, which was not discovered when each individual omics data was analyzed in isolation.

Consistent with this visualization, Fig. 3b shows the summary of pathways enriched in the subtype-specific subnetworks, drawn from the table generated by the software. The luminal B subtype had upregulation of estrogen receptor signaling, while luminal A subtype showed strong enrichment of downregulated cell cycle-related pathways. luminal B subtype showed enrichment of the hormone receptor and signal transduction pathways as well as FOXA1 and AP1 transcription regulatory network, indicating the protein-level evidence of ER signaling network is stronger in luminal B subtype than luminal A subtype in these data. As expected, HER2E subtype showed enrichment of upregulated signaling pathways that are not upregulated in the luminal subtypes, including epidermal growth factor receptor and innate immune response. The basal-like subtype largely showed upregulation of the PLK1 signaling cascade, APC/C-mediated degradation of cell cycle proteins, and small GTPase-mediated signal transduction, with characteristic downregulation of all hormone receptor-related pathways.

### Impact of DNA copy number-based normalization of mRNA data

We next compared the subnetworks reported by iOmicsPASS with normalization of mRNA data by DNA copy number data with the subnetworks acquired from the data without copy number-based normalization. If the user decides to normalize mRNA data by DNA copy number, this implies that each TF edge in the predictive networks is interpreted through the interaction scores of TF protein and its target gene’s mRNA per DNA copy (default option when all three omics data are available). If the user does not normalize the mRNA data by DNA copy number, then its interpretation becomes the interaction scores of TF protein and its target mRNA, regardless of the copy number variation. While the former option leads to the interpretation of interaction scores for TF edges with respect to the rate of transcription per DNA copy of a gene, it also requires generation of DNA copy number data and its inclusion may introduce additional noise into the combined data.

The analysis without DNA copy number data produced a smaller subnetwork at its optimal threshold (2578 edges, Supplementary Fig. 5), with a similar level of cross-validated error rates. When we compared the two subnetworks, the two analyses shared 1851 edges (see the analysis without normalization in Supplementary Table 2). The edges with larger centroid values were retained in both analyses, especially the PPI edges and the TF edges that are directly related to the hormone receptors such as ESR1 protein and ERBB2 protein. One major difference is that the predictive signature of luminal A subtype with DNA copy number normalization does not contain the TF network of ESR1 and its connection to FOXA1, as the subnetwork representing DNA damage response and repair was more densely connected and our scoring adjustment determined that the latter network was more predictive of subtype than the former. Moreover, the positive signature of CEBPB TF protein in the basal-like subtype was considerably weakened in the analysis without copy number-based normalization, which suggests that the normalization can help enhance the transcription regulation signature.

### Comparison of iOmicsPASS to other methods

Next, we compared the classification performance of iOmicsPASS in the BRCA data with comparison to other multiomics data integration approaches based on cross validation. Since we limit our feature selection space to the user-provided network, it is important to ensure that the predictive power is not compromised by the bounded feature search space. Our method is inherently a supervised classification method, but network-based supervised classification methods that can take multiomics data are scarce. Hence we compared iOmicsPASS to a few alternative tools available. We note that we attempted the nonlinear SVM classifier, yet the implementation in R did not finish the analysis within a few days (e1071 library), and thus we excluded the kernel learning algorithm in this comparison.

We first concatenated the two omics data sets into a data matrix and applied the NSC method. Supplementary Fig. 6 shows that the cross-validated misclassification rates of the NSC algorithm were slightly smaller than iOmicsPASS, especially for HER2E subtypes. However, when we examined the predictive features in the analysis, not only the method identified a large number of molecular features (4996 mRNAs, 170 proteins) as the predictive signature, 96.7% of the thousands of predictive nodes were mRNA molecules, and the number of selected features were above 5000.

In sum, despite seemingly better prediction performance, the concatenation-based integration coupled with the original NSC algorithm did not effectively incorporate additional predictive information provided by the proteomic data—the molecular level where the expression of functionally active gene products is observed. By contrast, the predictive subnetworks reported by iOmicsPASS analysis merged both molecular types in a more balanced manner, as all molecular interaction were forced to include protein-level information whether it is from the TF network or the PPI network.

We next explored the connection of iOmicsPASS to existing state-of-the-art multiomics data integration methods. As the majority of data integration approaches have been developed in an unsupervised analysis setting, we chose MOFA as a representative method for the class of LF analysis of multiomics data.

Interestingly, the MOFA analysis of the same mRNA-protein data set detected the first LF with prominent contribution from a large number of proteins. We found that this observation was an artifact of data quality issues in the proteomics data. As pointed out by Mertins et al. and the CPTAC,^17^ the iTRAQ ratios had aberrant global distributions for approximately a quarter of the samples (see Supplementary Fig. 7). We subsequently verified that LF2 and LF4 were the factors that separated the samples into the four intrinsic subtypes (Supplementary Fig. 8b).

Using the coordinates of samples in LF2 and LF4, we also identified that HER2E subtype did not form an independent cluster in this analysis. In fact, consistent with the iOmicsPASS analysis, many HER2E subjects were instead clustered with the luminal subtypes. We then considered the proteins with large loading scores on both LFs (LF2 and LF4) in the MOFA analysis. Although MOFA identified the key genes such as ESR1, FOXA1, GATA3, and AR in the top feature set, almost all top features in terms of the magnitude of loading scores were mRNAs, not proteins (Supplementary Fig. 8b). iOmicsPASS captured most of the same genes at the protein level since the method searches for predictive features within the bounds of interactions involving protein molecules, a prior that the user indirectly imposes in pursuit of predictive features.

## Discussion

In this work, we presented a supervised learning method for integrating multiomics data in the space of biological networks and extracting network signatures predictive of each phenotypic group in supervised analysis. The key difference of iOmicsPASS framework is that the predictive features are searched within the given set of molecular interactions and the scoring algorithm favors densely connected subnetworks enriched with predictive signals. Despite the constraints, iOmicsPASS was able to extract key interaction signatures without compromising its prediction performance in both simulated data and BRCA data, while the original NSC algorithm showed consistently poor classification performance in simulation data and picked heavily mRNA-centric gene signatures in the BRCA data.

An important advantage of iOmicsPASS is that the selected predictive signature forms densely connected subnetworks, rather than molecules that are scattered across the network. In other words, the method forcefully limits the search space of predictive features to the known interactions, and by doing so, the user essentially specifies a biological prior in the predictive feature selection. We showed that this framework was able to identify biological networks specifically relevant to subtype prediction without sacrificing prediction error rates. This guided analysis approach may provide advantages especially in data sets with a relatively modest sample size (e.g., tens of samples per phenotypic group), where the most advanced kernel learning algorithm failed to find clear decision boundaries.

Meanwhile, the method has room for future improvement. First, the current implementation does not provide functionalities for prediction of phenotypic groups in external data sets yet. This was a deliberate choice, since it is difficult to expect molecular profiling studies with all three omics platforms for hundreds of tumor samples, especially when MS-based proteomics is a part of the omics repertoire. Hence our immediate future work is to develop a prediction module to make phenotype predictions for a new data set with incomplete multiomics data. Second, as we integrate more diverse types of omics data, some omics data will yield more influence than others. As such, future work will explore optimal weighting of different omics data sets in the predictions. Third, our current implementation discards molecules that are not represented in the user-provided network data. Hence important markers that are poorly represented in biological networks can be lost in the analysis. Our future development will consider maintaining these predictive ‘singleton’ nodes. Lastly, and most importantly, future versions of iOmicsPASS will make probabilistic predictions that allow multiplicity in assignments. As we demonstrate in the examples above, there will be a subset of tumor samples that share characteristics of multiple subtypes, given typical heterogeneity in tumors. Hence a prediction method that allows for this possibility will provide a more biologically realistic framework than the current mutually exclusive subtyping. iOmicsPASS will be one of the first methods that systematically address this issue in prediction.

## Methods

### Calculation of interaction scores for biological networks

The first module of iOmicsPASS computes interaction score for each interaction represented in a user-provided biological network. For the integration of mRNA transcript and protein data, two types of networks are relevant: (1) PPI network to link different proteins and (2) TF regulatory network to link TF proteins with the mRNAs of their target genes. DNA copy number can also be incorporated as a normalizing constant for mRNA abundance, since the ratio of mRNA to DNA copy number can be considered as the “output” of gene transcription per DNA copy, i.e., transcription efficiency.

For the derivation of interaction scores in the context of mRNA and protein data integration, we let *p* denotes the total number of edges, *n* the number of samples, and *t* the type of interaction data (*t* = 1 for TF regulatory network, *t* = 2 for PPI network). For *i* = 1,…,*p*, *j* = 1,…,*n*, and *t* = 1 or 2, the interaction score for edge *e*_*ijt*_ is calculated as:

When DNA copy number data are not provided,

1 $$e_{ij1} = z_{{\rm{prot}}_A,j} + z_{{\rm{mRNA}}_B,j},$$

2 $$e_{ij2} = z_{{\rm{prot}}_A,j} + z_{{\rm{prot}}_B,j}.$$

When DNA copy number data are provided,

3 $$e_{ij1} = z_{{\rm{prot}}_A,j} + \left( {z_{{\rm{mRNA}}_B,j} - z_{{\rm{dna}}_B,j}} \right),$$

4 $$e_{ij2} = z_{{\rm{prot}}_A,j} + z_{{\rm{prot}}_B,j},$$

here *z* represents the *Z*-score of log-transformed measurement (base 2) of each molecule in the respective omics data sets, and hence addition and subtraction of these *Z*-scores are equivalent to multiplication and division of the original abundance data in the original scale. When the type of edge is created from a TF regulatory network (i.e., *t* = 1), prot_*A*_ represents the protein of a TF gene A, and mRNA_*B*_ is the mRNA of target gene B. dna_*B*_ refers to the DNA copy number of the same gene (target gene B). On the other hand, when the type of edges is PPI network (i.e., *t* = 2), prot_*A*_ and prot_*B*_ represent the proteins of the respective genes A and B. From here on, we use the term “feature” to refer to edges or their interaction scores.

### Subnetwork discovery module

Using the feature data created above, iOmicsPASS identifies a sparse subset of interactions whose interaction scores are predictive of phenotypic groups. iOmicsPASS applies the NSC method, originally introduced in the Prediction of Analysis of Microarray method,^13^ to interaction scores. The NSC algorithm is known to have bias in assigning samples to groups of a larger sample size,^18,19^ which motivated us to extend the original algorithm in several ways, to account for the dependence between features and the selection bias in sampling.

First, we computed the centroid for feature *i* considering the sample size of each phenotypic group (e.g., tumor subtypes). Specifically, we compute it as

5 $$\bar x_i = \frac{1}{K}\mathop {\sum }\limits_{k = 1}^K \bar x_{ik} = \frac{1}{K}\mathop {\sum }\limits_{k = 1}^K \left( {\frac{1}{{n_k}}\mathop {\sum }\limits_{j \in C_k} x_{ij}} \right),$$

where *C*_*k*_ represents the indices of *n*_*k*_ samples in phenotypic group *k* and *K* is the number of phenotypic groups. This calculation avoids the sampling bias towards the phenotypic groups with large sample sizes.

Second, in the NSC method, *d*_*ik*_ is defined to be the *t*-statistic for each gene *i*:

6 $$d_{ik} = \frac{{\bar x_{ik} - \bar x_i}}{{m_k\,(s_i + s_0)}},$$

where $\bar x_{ik}$ represents the centroid of gene *i* in phenotypic group *k* and $\bar x_i$ represents the overall centroid of gene *i*. The denominator serves as a normalizing constant, where $m_k = \sqrt {1/n_k + 1/n}$, *s*_*i*_ is the pooled within-class standard deviation of gene *i*, and *s*_0_ is the median value of *s*_*i*_ across all features, a positive constant.

In iOmicsPASS, instead of genes, the term *d*_*ik*_ refers to the test statistic for edge *i* in phenotypic group *k*. We add an extra term to *d*_*ik*_, which accounts for the consistency of interaction scores in neighbor edges, i.e., edges that share common nodes with the current edge *i*. For every edge *e*_*i*_, we define neighbor edges of *e*_*i*_ as the ones that share at least one of the two nodes with it. We now define a new test statistic for edge *i*, $d_{ik}^ \ast$, as:

7 $$d_{ik}^ \ast = d_{ik} + \left( {\psi _{i,k} \times \frac{{|N_{e_i,1}\,|\mathop {\sum }\nolimits_{s \in N_{e_i,1}} d_{sk} + |N_{e_i,2}\,|\mathop {\sum }\nolimits_{r \in N_{e_i,2}} d_{rk}}}{{\left| {N_{e_i}} \right|}}} \right),$$

where $N_{e_i}$ represents the set of neighbor edges of *e*_*i*_, and $|N_{e_i}|$ denotes the number of edges in the neighborhood set $N_{e_i}$. The set $N_{e_i}$ is further partitioned into two subsets, $N_{e_i,1}$ and $N_{e_i,2}$, to represent the set of TF and PPI edges, respectively. The multiplicative factor $\psi _{i,k}$ for phenotypic group *k* represents the proportion of agreement in sign (direction of change) between *e*_*i*_ and its neighbor edges. Specifically, it is calculated by:

8 $$\psi _{i,k} = \frac{{2e^{5\left( {p_{ik} - 0.5} \right)}}}{{1 + e^{5\left( {p_{ik} - 0.5} \right)}}},$$

where

9 $$p_{ik} = \frac{{\mathop {\sum }\nolimits_{j \in N_{e_i},i \ne j} {\mathrm{sign}}\left( {d_{ik}} \right) = {\mathrm{sign}}\left( {d_{jk}} \right)}}{{|N_{e_i}|}}.$$

We tested other variants of this function and the functional form did not impact the shape of the subnetworks significantly as long as its range is between 0 and 2, and it is greater than 1 if at least half of the neighbor edges have consistently up- or down-regulated interaction scores with that of edge *e*_*i*_. These adjustments ensure that the local subnetworks densely populated by predictive edges with identical sign are favored to the ones with predictive edges scattered randomly.

Third, for each phenotypic group *k*, a group-specific threshold Δ_*k*_ is derived from the shrinkage parameter, Δ, calculated by:

10 $$\Delta _k = \frac{\Delta }{{\Delta _{{\rm{max}}}}} \times {\rm{max}} _i\,d_{ik},$$

where

11 $$\Delta _{{\rm{max}}} = \frac{1}{K}\mathop {\sum }\limits_{k = 1}^K {\rm{max}} _i\,d_{ik}.$$

We set the values of Δ on a grid of 30 equally spaced values arranged in an increasing order (i.e., $\Delta \in \{ 0,\Delta _1,\Delta _2, \ldots ,\Delta _{{\mathrm{max}}}\}$) from zero to a value sufficiently large such that the group-specific centroids of all the features are reduced towards the overall centroid as Δ increases. Using a soft-thresholding approach, each feature’s score is recomputed as $d_{ik}^\prime$, reducing it by an absolute shrinkage amount incrementally, until it reaches zero:

12 $$d_{ik}^\prime = {{\rm{sign}}}\left( {d_{ik}^ \ast } \right)\left( {\left| {d_{ik}^ \ast } \right| - \Delta _k} \right)_ +,$$

where $\left( {\left| {d_{ik}^ \ast } \right| - \Delta _k} \right)_ + = \max \left( {0,\left| {d_{ik}^ \ast } \right| - \Delta _k} \right)$and ${{\rm{sign}}}\left( {d_{ik}^ \ast } \right) = 1$ if $d_{ik}^ \ast > 0$, ${{\rm{sign}}}\left( {d_{ik}^ \ast } \right) = - 1$ if $d_{ik}^ \ast < 0$ and ${{\rm{sign}}}\left( {d_{ik}^ \ast } \right) = 0,$ otherwise. We choose the optimal value for Δ based on cross validation to yield a sparse set of predictive features. Nonzero score (i.e. $|d_{ik}^\prime | > 0$) indicate that the feature has certain level of predictiveness and will be used to form a subnetwork that best classifies samples to their phenotypic groups using the discriminant scores defined below.

Suppose that we have a test sample with edge-level interaction scores $x_1^ \ast = \left( {x_1^ \ast ,x_2^ \ast , \ldots ,x_p^ \ast } \right),$ then we compute the discriminant score for group *k* as:

13 $$\delta _k\left( {x^ \ast } \right) = \mathop {\sum }\limits_{i = 1}^p \frac{{\left( {x_i^ \ast - \bar x\prime _{ik}} \right)^2}}{{\left( {s_i + s_0} \right)^2}} - 2\log \left( {\pi _k} \right),$$

where *π*_*k*_ is the prior probability of the *k*^th^ class (equal prior *π*_*k*_ = 1/*K* by default). Then we assign each test sample to the phenotypic group with the smallest discriminant score using the classification rule: $C\left( {x^ \ast } \right) = \ell$ where $\delta _\ell \left( {x^ \ast } \right) = \mathop {{\min }}\limits_k \delta _k\left( {x^ \ast } \right)$. Using the discriminant scores, the estimated probability of sample x^*^ membership to group *k* is computed as:

14 $$\hat p_k\left( {x^ \ast } \right) = \frac{{e^{ - \frac{1}{2}\delta _k\left( {x^ \ast } \right)}}}{{\mathop {\sum }\nolimits_{\ell = 1}^K e^{ - \frac{1}{2}\delta _\ell \left( {x^ \ast } \right)}}}.$$

### Pathway enrichment module for subtype-specific networks

Lastly, iOmicsPASS tests enrichment of biological functions and pathways in the selected subnetwork for each phenotypic group. We first separate the edges into those with positive and negative $d_{ik}^\prime$ scores, separately for each phenotypic group. Then, we apply hypergeometric test to compute the probability of overrepresentation of those edges in pathways. We set all possible edges in the user-provided network that were available in the data as the background. See Supplementary Methods for details.

### Multiomics data, pathways and biological networks

Both breast data were downloaded using Genomics Data Commons (GDC) data portal from TCGA and proteomics data were downloaded from the CPTAC.^20^ The transcriptomic and copy number variation data were processed using GDC processing pipelines (with GRCh38 as the reference genome).

To standardize the type of gene identifiers used across the different omics data, all identifiers were mapped to HGNC gene symbol. ENSEMBL identifiers were converted to gene symbols in the mRNA data and gene symbols were already provided in the proteomics data. For CNV data, the chromosomal positions were used to infer the genomic coordinates and a gene symbol was assigned. If two or more genes overlap at the start and end site, a weighted average of the segment mean values was computed for each gene where the weights are proportional to the length of the segment occupied. Then, the average segment mean was computed for each gene in each individual to obtain a segmental mean value per gene.

We downloaded two types of biological networks: PPI network and TF regulatory network. For the PPI network, the two sources were from iRefIndex^21^ and BioPlex 2.0,^22^ where we removed the redundancies and compiled into an integrated database. For the TF regulatory network, we collected the interactions between (TF) proteins and target genes and put together from the following sources: TRED,^23^ ITFP,^24^ ENCODE, and TTRUST.^25^ The final network consisted of 16,266 proteins forming 197,664 edges in the PPI network and 2486 TFs and 14,796 target genes forming 101,272 edges in the TF network.

For the pathway data, we used biological pathways from the ConsensusPathDB (CPDB)^26^ and Gene Ontology (GO).^27^ The pathways from the CPDB include data from multiple sources such as KEGG, PharmGKB, SMPDB, HumanCyc, BioCarta, EHMN, Reactome, NetPath, Pathway Interaction Database, and Wikipathways. For GO, we considered the biological processes only. As a result, the final pathway consisted of a total of 14,598 pathways involving 17,250 genes. All network and pathway files are distributed along with the tool.

## Supplementary information

Supplementary Information

## Data Availability

iOmicsPASS is an OS platform-independent tool, written in C++ language. It is freely available through GitHub repository at https://github.com/cssblab/iOmicsPASS. The tool is distributed in a zip folder, along with sample data sets and a software manual. Supplemental R codes are provided for the visualization of misclassification errors (cross validation) and for the choice of appropriate thresholds. The processed data sets (copy number variation, mRNA and protein expression data) used for breast cancer data from TCGA, as well as R codes used to produce the heatmaps and plots in this paper can be downloaded from https://github.com/Hiromikwl/DataCodes_iOmicsPASS. The output of the tool used for network visualization in Cytoscape is provided as Supplementary Tables, which are freely available at *NPJ Systems Biology and Applications website*. The R codes to generate simulation data sets are also provided through the GitHub site.

## Supplementary information

**Supplementary Information** accompanies the paper on the *npj Systems Biology and Applications* website (10.1038/s41540-019-0099-y).
