# Supplementary material for: iOmicsPASS: network-based integration of multiomics data for predictive subnetwork discovery
Source: NPJ Syst Biol Appl. 2019 Jul 9;5:22. doi: 10.1038/s41540-019-0099-y (PMC6616462; doi:10.1038/s41540-019-0099-y)
Supplement: Supplementary file 1 — Supplementary Information [file 41540_2019_99_MOESM1_ESM.docx]

**Supplementary Information for “iOmicsPASS: network-based integration of multi-omics data for predictive subnetwork discovery**”

Hiromi W.L. Koh^1,2^, Damian Fermin^3^, Christine Vogel^4^, Kwok Pui Choi^5^, Rob Ewing^6^,

Hyungwon Choi^1,2,7,*^

^1^ Department of Medicine, Yong Loo Lin School of Medicine, National University of Singapore

^2^ Saw Swee Hock School of Public Health, National University of Singapore, Singapore

^3^ University of Michigan Medical School, Ann Arbor, MI, USA

^4^ Center for Genomics and Systems Biology, Department of Biology, New York University

^5^ Department of Statistics and Applied Probability, National University of Singapore, Singapore

^6^ School of Biological Sciences, University of Southampton, Southampton, United Kingdom

^7^ Institute of Molecular and Cell Biology, Agency for Science, Technology and Research, Singapore

* To whom all correspondence should be addressed. E-mail: hwchoi@nus.edu.sg

**Supplementary Methods**

1. **Pathway enrichment analysis for subnetworks**

Here we describe the edge count-based hypergeometric test. Given a graph $G = (V, E)$where $V$ is the set of all possible nodes (i.e. mRNA and proteins available in the data) and $E$ is the set of all edges (i.e. TF and PPI interactions from network information) in the graph. We can construct smaller subgraphs, $G_{p} = (V_{p},E_{p})$ to represent every pathway where $V_{p}$ (i.e. $V_{p}\subset V$ ) is the set of nodes representing all molecules in pathway $p$ and $E_{p}$ (i.e. $E_{p}\subset E$) is the set of all edges in the network that connects $V_{p}$. Using the set of selected edges for each phenotypic group $k$, we create an induced subgraph of $G_{p}$, $G_{k} = (V_{k},E_{k})$, where $V_{k}$ (i.e. $V_{k}\subseteq V_{p}$) is the set of nodes connected by the selected edges and in pathway $p$, $E_{k}$ (i.e. $E_{k}\subseteq E_{p}$) is the set of all edges present in pathway $p$ and $V_{k}$.

Then we calculate the probability of over-representation of selected edges in a particular pathway from hypergeometric distribution. We consider $X=|E_{k}|$ as a hypergeometric random variable

$$X \sim Hypergeometric(K,N, n)$$

$$Pr\left( over representation \right)= Pr\left( X > x \right)= 1- Pr\left( X\leq x \right),$$

where $K$is the number of edges in the selected subnetwork$, N$is the total number of edges in the entire network (background), and $n=|E_{p}|$ is the number edges connecting the nodes that belong to the pathway of interest., In the analysis presented in the paper, we only consider the pathways with at least 3 edges in the selected subnetwork.

1. **Simulation study**

We randomly sampled 1,000 TF proteins and 5,000 mRNAs from the original network data, where all the proteins are transcription factors and the latter are targets of those transcription factors. One hundred sets of quantitative proteomic and transcriptomic data were simulated, assuming that the data was collected from 50 subjects in a disease group and 50 control subjects. We planted the signal, i.e. non-zero centroid in 100 randomly selected TF proteins and in the mRNAs of all of its interacting partners with stipulated probabilities among the samples of the disease group.

To make the exercise realistic, we applied a probability called *assay sensitivity* (i.e. *P_AS_*) to determine how likely protein-level signals are captured by the MS data, i.e. to account for the possibility of loss of signal during the assay quantification step such as mass spectrometry-based proteomics experiments (**Supplementary Figure 1A**). Protein measurements in the disease group were generated from Gaussian distribution with mean, $\mu,$ and standard deviation, $\sigma$ with probability *P_AS_*; while those in the control group were generated from Gaussian distribution with zero mean and the same standard deviation. Likewise for the mRNA data, mRNA measurements in the disease group were generated with probability *P_mRNA_*, the probability that TF has a real impact on the transcription of the mRNA, proportional to the number of transcription factor targeting it (parent nodes) (see **Supplementary Figure 1B**). Hence, mRNAs with a greater number of TFs targeting itself will have a higher probability of carrying a differential signal.

Another consideration in the simulation was that the homeostasis of the proteome is generally more tightly controlled than that of the transcriptome in dynamic cellular environment.^1^ To reflect the empirical observation that typical proteomic data shows more attenuated fold changes than transcriptomic data between different phenotypic groups, we also considered stronger signals in the mRNA data than in the protein data in one of the simulation setups (Setup C).

Simulations were carried out using three different settings: (**A**) signal in the disease group is the strongest with *μ*_Protein_ = *μ*_mRNA_ = 1 and *σ*_Protein_ = *σ*_mRNA_ = 1; (**B**) signal is noisier with *μ*_Protein_ = *μ*_mRNA_ = 0.5 and *σ*_Protein_ = *σ*_mRNA_ = 1; and **(C**) attenuated signal in protein data compared to mRNA data with *μ*_Protein_ = 0.5, *μ*_mRNA_ = 1 and *σ*_Protein_ = *σ*_mRNA_ = 1.

Specifically, we carried out the simulations as follows:

1. Randomly select 100 TF proteins to be the true signals.
2. Identify mRNA targets of the 100 TF proteins to plant signals in.
3. Generate quantitative data for proteins and mRNAs as follows. For each molecule *i* in sample *j*, simulate as follows:
   1. For protein data:
      1. Samples in the disease group are generated with probability *P_AS_*,
         *X_ij_ ~ N(μ*, *σ*^2^).
      2. Samples in the control group are generated by *X_ij_ ~ N(*0, *σ*^2^).
   2. For mRNA data:
      1. Samples in the disease group are generated with probability, *P_mRNA_*,
         *X_ij_ ~ N(μ*, *σ*^2^).
      2. Samples in the control group are generated by *X_ij_ ~ N(*0, *σ*^2^).
4. Perform iOmicsPASS analysis on the integrated interaction scores with and without network-oriented scoring adjustment and NSC analysis on the node-level data after concatenating both data together into a single matrix (using PAMR package in R).

The simulations were repeated 100 times and sensitivity and specificity were averaged across the data sets to produce the average ROC curve. Both sensitivity and specificity were calculated at the edge level, where the true signal is defined to be an edge that encompasses both the 100 TF proteins and the mRNAs of their target genes (e.g. interacting proteins or mRNAs targets). For the NSC algorithm, using the results generated by PAMR, edges were constructed from the set of nodes that are selected. Hence, both the protein and mRNA must be selected in the centroid for the edge to be considered as selected. Sensitivity was calculated using number of true edges selected in each method, divided by the number of true edges. Specificity was the number of edges without signal and not selected in each method, divided by the number of edges without a signal. Using the set of sensitivities and specificities computed across multiple thresholds, the area under the curve (AUC) is then calculated using the trapezoidal rule.

**Simulation with incomplete network**

So far the key assumption in the simulation is that we know all “true” interactions between molecules. In real applications, the network data is incomplete, carrying false positives and false negatives. We simulated *incomplete networks* that include spurious interactions and/or lack a portion of true interactions in order to test the robustness of iOmicsPASS in these situations. We simulated the incomplete networks and data in two different ways. In the first approach, we added and removed edges randomly; in the second approach, we removed nodes on the network randomly to reduce the size of the network.

***Approach 1: Incomplete network via removal and addition of edges***

In this setup, we modify the *complete network* by removing randomly selected edges or adding random edges. Edges can only be added between every pair of nodes that do not have an edge between them. Assuming that there are $n$nodes in the network, we can form a complete graph ($K_{n}$) with $\frac{n(n-1)}{2}$ number of possible edges. Then, the number of possible edges that we can add is$\frac{n(n-1)}{2}-p$, where *p* the number of edges in the *complete network*. Here, we define two types of coins with biased probability of heads: C_1_ (i.e. C_1_=1) with probability 0.1 and C_2_ (i.e. C_2_=1) with probability 0.0025. The latter probability was suggested to balance the number of additions and removals, i.e. to keep the overall network size about the same as the complete network.

The detailed steps are as follows:

1. For every edge $e_{i}$ in the network, we toss the first coin C_1_.
   1. If C_1_=1, we remove $e_{i}$ from the network.
   2. Else, we keep$e_{i}$.
2. Then, for every pair of nodes without an edge between them, we toss the second coin C_2_.
   1. If C_2_=1, we add an edge between them.
   2. Else, continue.

***Approach 2: Incomplete network via removal of nodes***

In this setup, we remove parts of the *complete network* by removing randomly selected nodes. We again define a biased coin (C) with probability of heads (i.e. C=1) 0.9 and tail (i.e. C=0) 0.1.

Then we create the network as follows:

1. For every node $v_{j}$ in the network, we toss the coin (C).
   1. If C=1 (i.e. heads), we keep $v_{j}$ in the network.
   2. Else, we remove $v_{j}$ from the network.
2. We form a smaller network induced by the set of sampled nodes and treat them as a new network.

***Prediction Performance using Incomplete Network***

In the complete network, 48,682 edges and 6,742 edges (13.8%) are defined to be the *true* *edges* with differential signal. In the first approach, we generated an incomplete network by removal and addition of edges (**Supplementary Figure 2A**). We removed a total of 4,891 edges and added 12,416 spurious interactions to the complete network. The resulting network had a total of 56,207 edges where 10.8% were carrying signal. However, 689 (10.2%) true edges were lost from the network.

In the second approach, we created a smaller network by removing nodes from the complete network. We removed 38 proteins and 241 mRNAs, which account for 4.7% of the nodes in the complete network. As a result, we removed 9,221 edges where 14.0% were carrying signal, and 1,295 (19.2%) of the true edges were lost from the network.

**Supplementary Figure 2B** shows the receiver-operating characteristic (ROC) curves for the three simulation setups when using probability of assay sensitivity (P_AS_) of 70%. Overall, iOmicsPASS analysis with incomplete network resulted in poorer sensitivity and specificity than the analysis with the complete network. However, the overall prediction performance is still better than the node-level analysis using the NSC algorithm and almost equivalent in simulation setup C where the difference in AUC between the NSC algorithm and iOmicsPASS with the complete network was the smallest. Even though the ROC curves using an incomplete network (i.e. blue and green dotted lines) eventually crosses the ROC curve for the NSC algorithm when specificity reduces to below 50%, the threshold is usually selected at a point with high specificity (to the left) before the crossing occurs. We also repeated the exercise using assay sensitivity probability (P_AS_) of 80% and the results remained similar (not shown).

**Supplementary Figures**

**
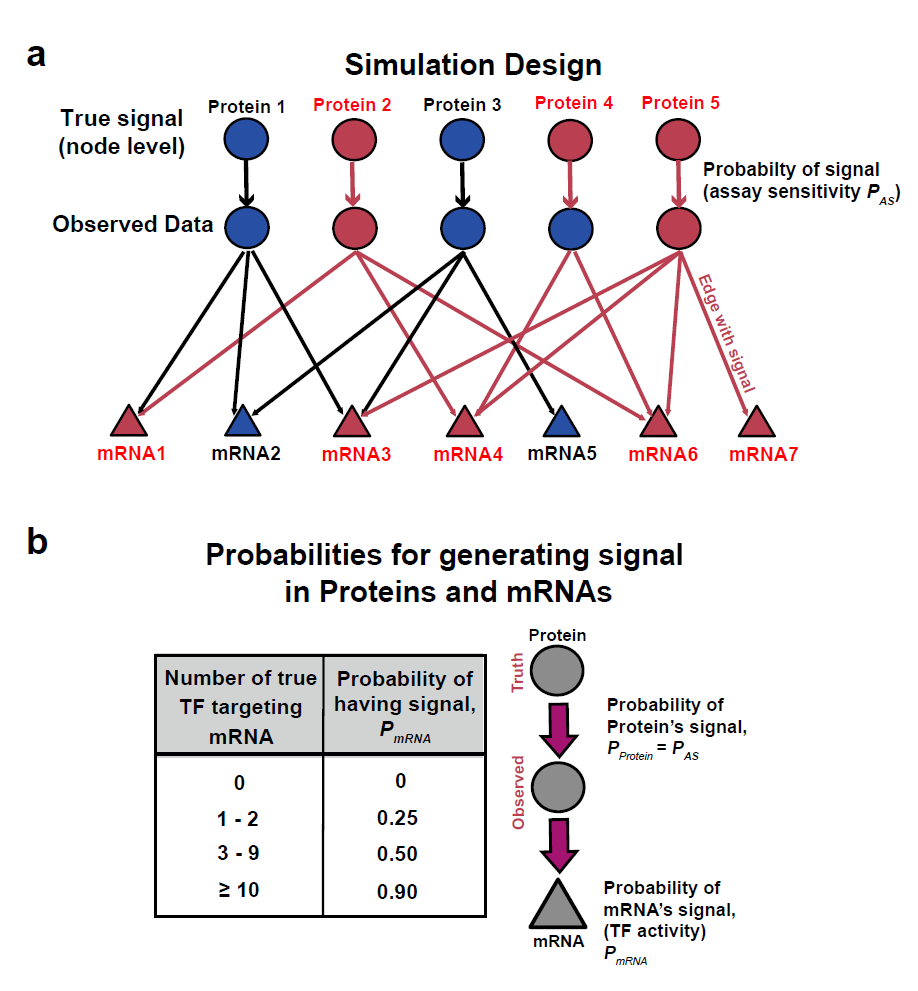
**

**Supplementary Figure 1.** Simulation settings and results. (**a**) To reflect the noise level of mass spectrometry-based proteomics, we simulate the protein-level data with a parameter called assay sensitivity (*P_AS_*), i.e. the probability that the quantitative proteomic data detects differential signal in a protein. (**b**) We set a varying probability of mRNA molecules having a differential signal based on the number of active (more abundant) TF proteins targeting each gene (*P_mRNA_*).


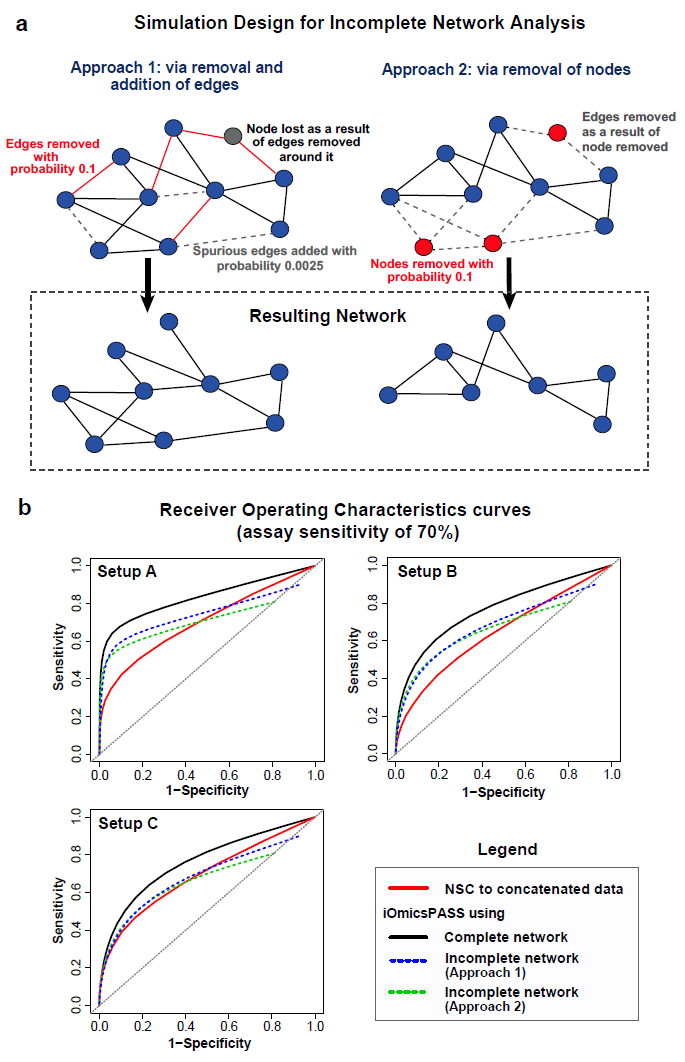


**Supplementary Figure 2**. Simulation settings and results for the analysis with incomplete networks. **(a)** The simulation design for generating an incomplete network using two types of approaches: *Approach 1*- via removal and addition of network edges to produce a noisy network and *Approach 2* - via removal of network nodes to induce a smaller network data. **(b)** The Receiver Operating Curves for the NSC algorithm (concatenated data) and iOmicsPASS (interaction scores) under three different signal-to-noise ratio settings. Red solid line denote the performance of the NSC algorithm, black solid lines denote the performance of iOmicsPASS analysis using *complete network* and the blue and green dotted lines show the performance using iOmicsPASS using *incomplete network* using the two approaches described in **(a)**, respectively.

**
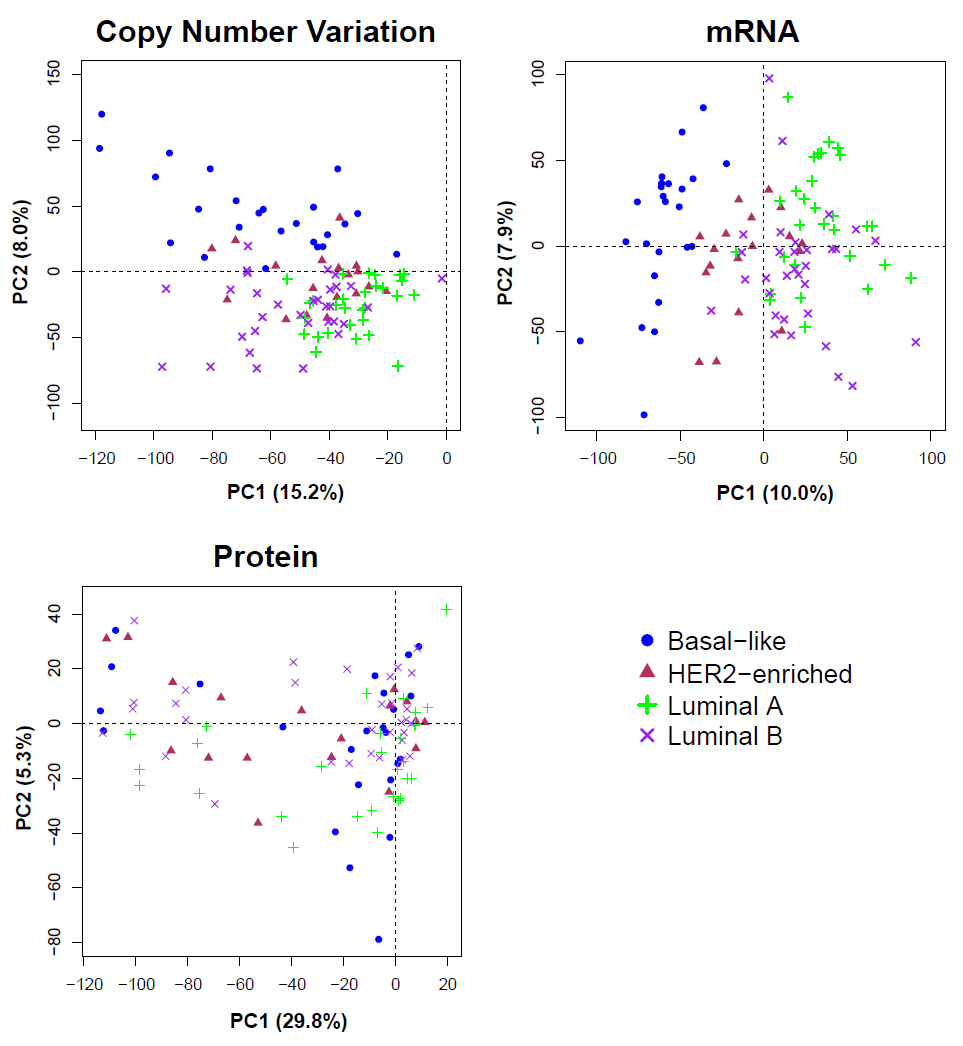
**

**Supplementary Figure 3.** PCA plots of the three -omics data sets from the TCGA BRCA data. Proteomics data show indication of sample variation unrelated to the intrinsic subtypes. The difference in proteomic profile between the observations with PC1 scores below -50 those above -50 are visualized separately in the MOFA analysis (**Supplementary Figure 7**.)

**
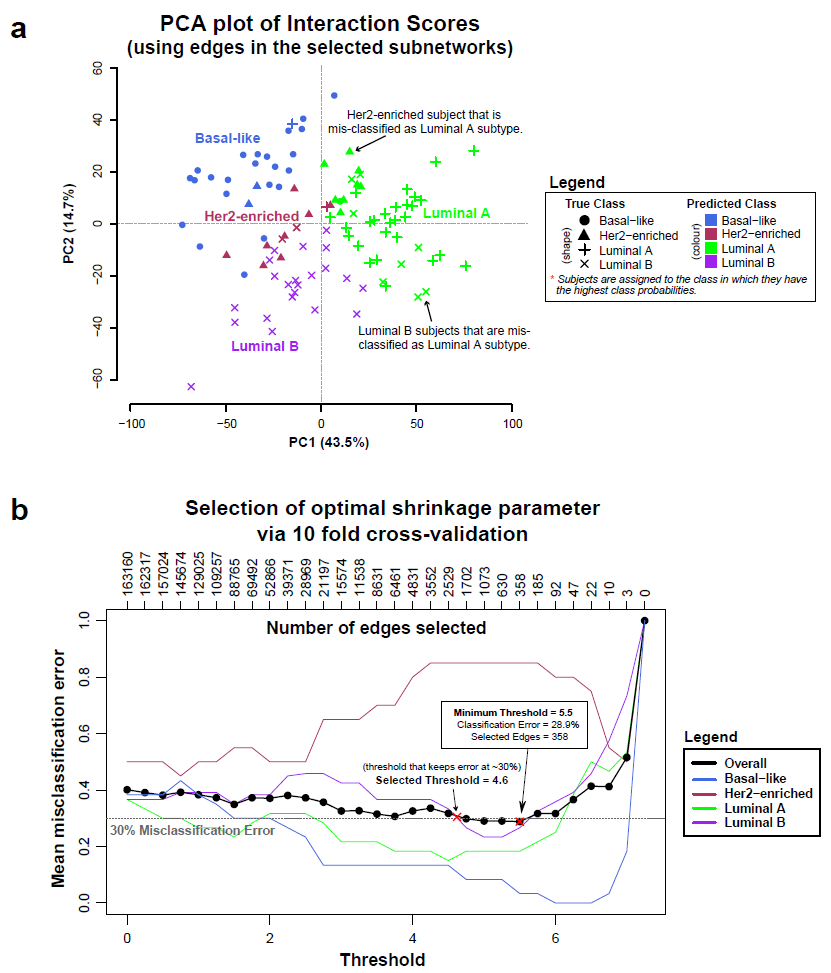
**

**Supplementary Figure 4.** (**a**) PCA plots of the interaction scores for the selected subnetwork in BRCA data (not the entire data). True class (mRNA-based PAM50 ^2^) and predicted class (by the optimal prediction model within the training data) are indicated by different shapes and colors, respectively. (**b**) Misclassification errors (test errors) computed for each subtype using 10-fold cross-validation. The red color cross symbol on the right side is the threshold that gives the smallest misclassification error rate and the sparsest network, and the one on the left side is the threshold giving a less sparse subnetwork. We chose the left threshold considering that each class is represented by a small number of samples (~25 per group). The standard practice should adhere to the selection of sparser networks in large-sample data sets.

**
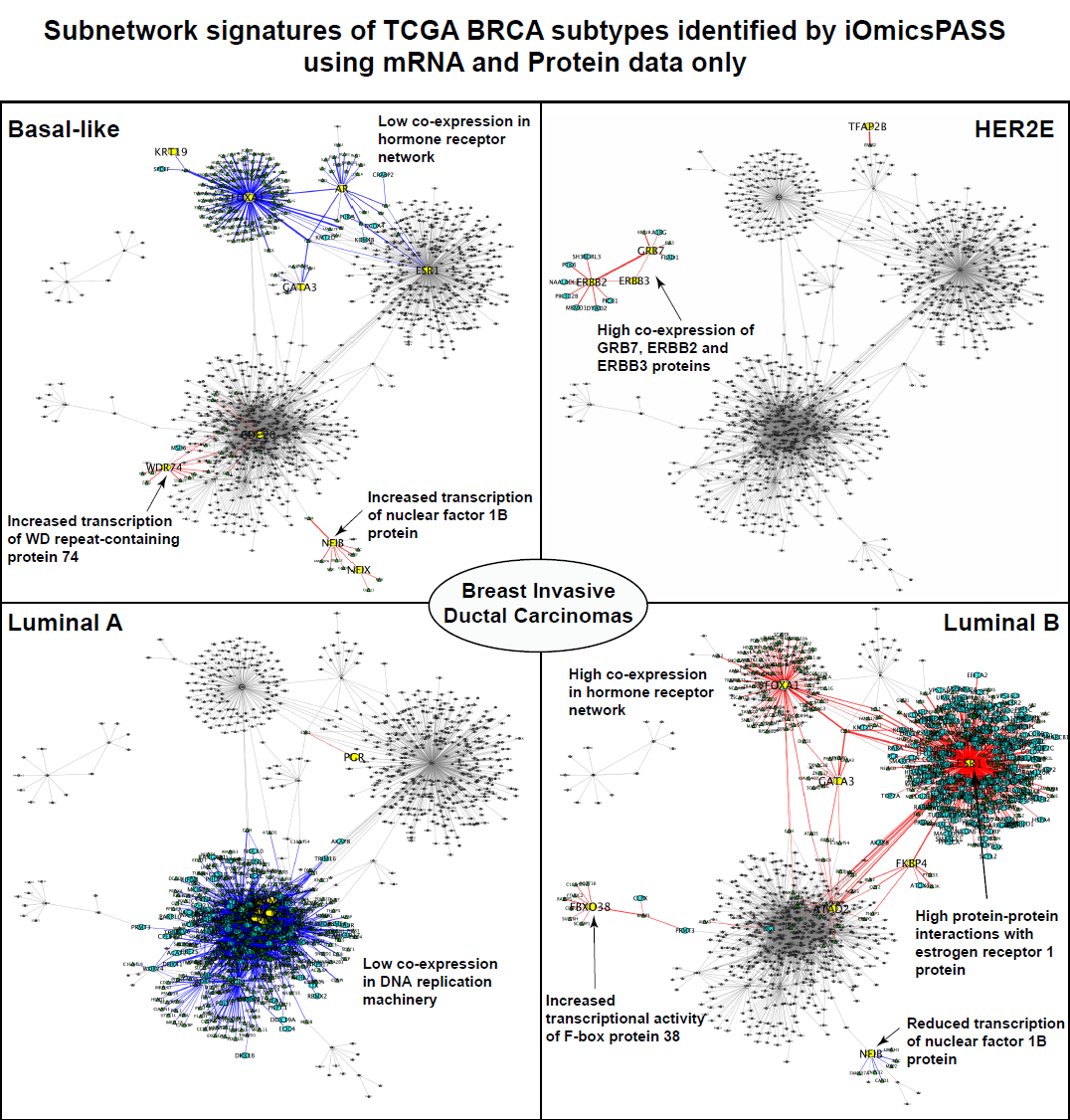
**

**Supplementary Figure 5.** Selected predictive subnetworks in the analysis without DNA copy number-based normalization of mRNA data and without scoring adjustment. This analysis is equivalent to the NSC algorithm applied to the interaction scores. In comparison to **Figure 4**, there are two major differences. First, Basal-like subtype has weaker representation of up-regulation in CEBPB and NFIB TF network. Second, estrogen receptor signaling is part of a Luminal A subtype predictive signature (red edges on the left side), which was removed in the analysis with copy number normalization and scoring adjustment (**Figure 4**).

**
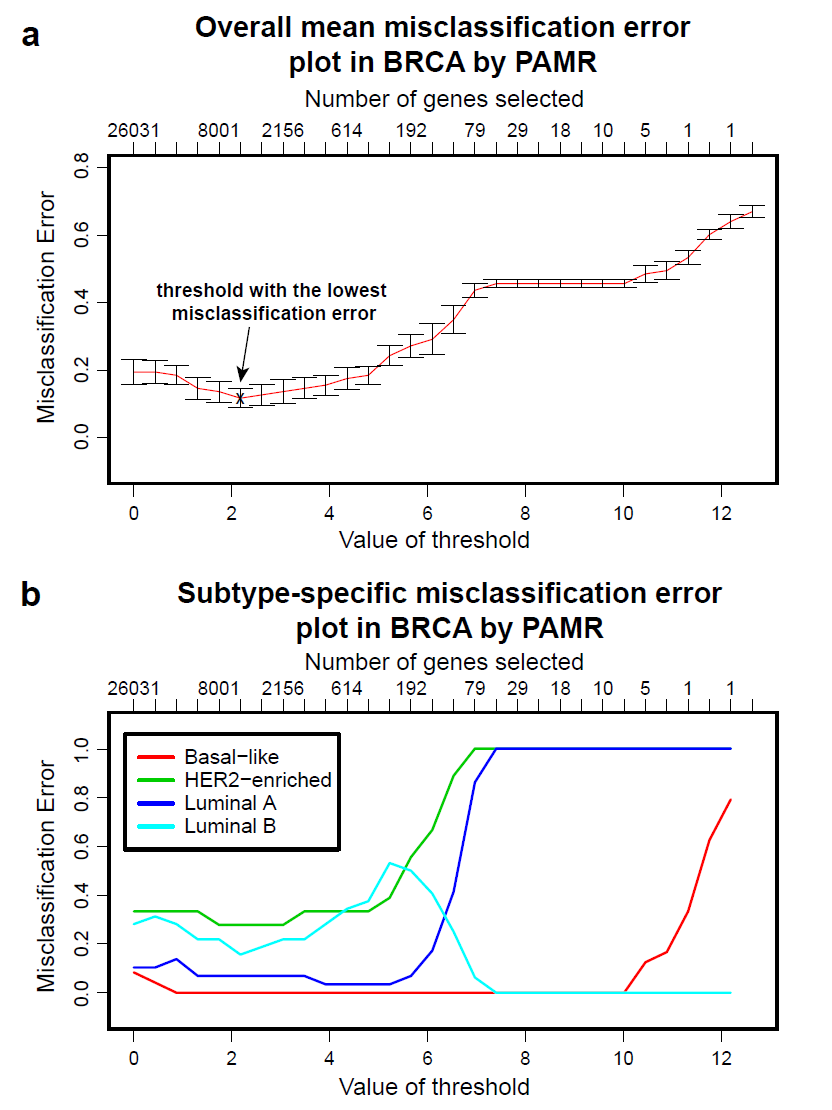
**

**Supplementary Figure 6**. (**a**) Cross-validated misclassification error rates in the NSC algorithm (PAMR package) applied to the concatenated data in TCGA BRCA. The optimal model contained 5,166 features (4,996 mRNAs and 170 proteins) (**b**) Class-specific misclassification error rates.


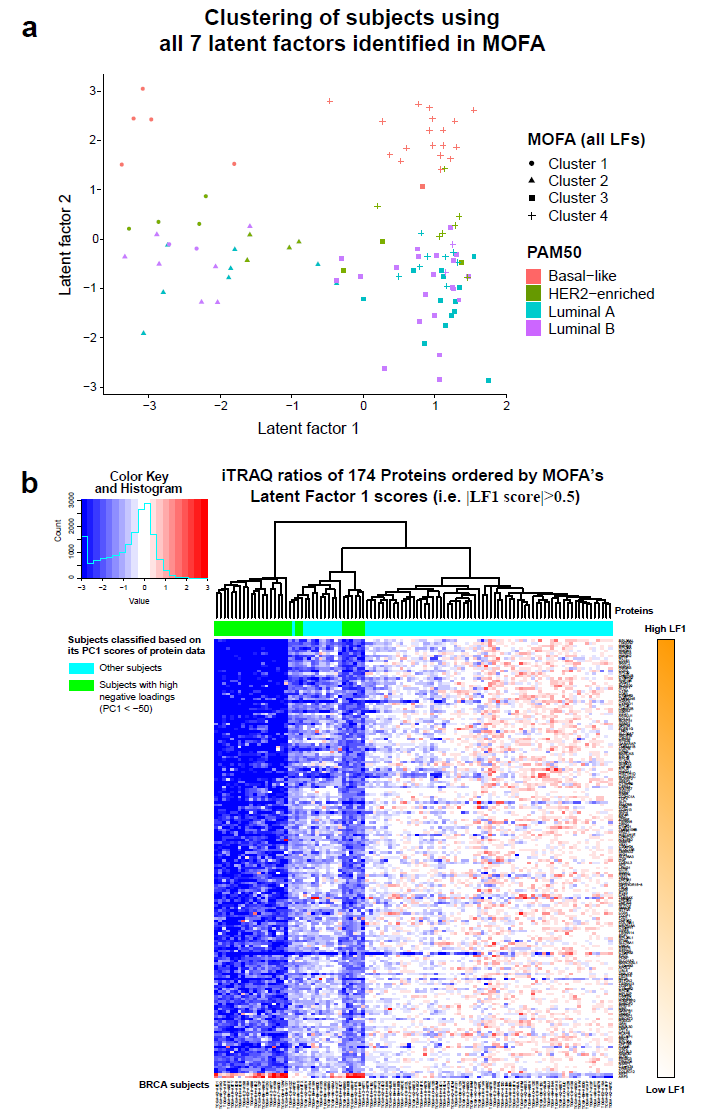


**Supplementary Figure 7**. (**a**) Plot of latent factor scores (factors 1 and 2) from the MOFA analysis. MOFA analysis suggested a total of 7 factors present in the data. Consistent with the unwanted noise in the proteomic data, MOFA’s latent factor 1 was mainly driven by the variation in the proteomics experiment. (**b**) Protein profile with the top scores on latent factor 1, with a color bar indicating the PC1 scores in **Supplementary Figure 3**. As reported in *Mertins et al*.^3^, proteomic data in about a quarter of the subjects had quality issues.


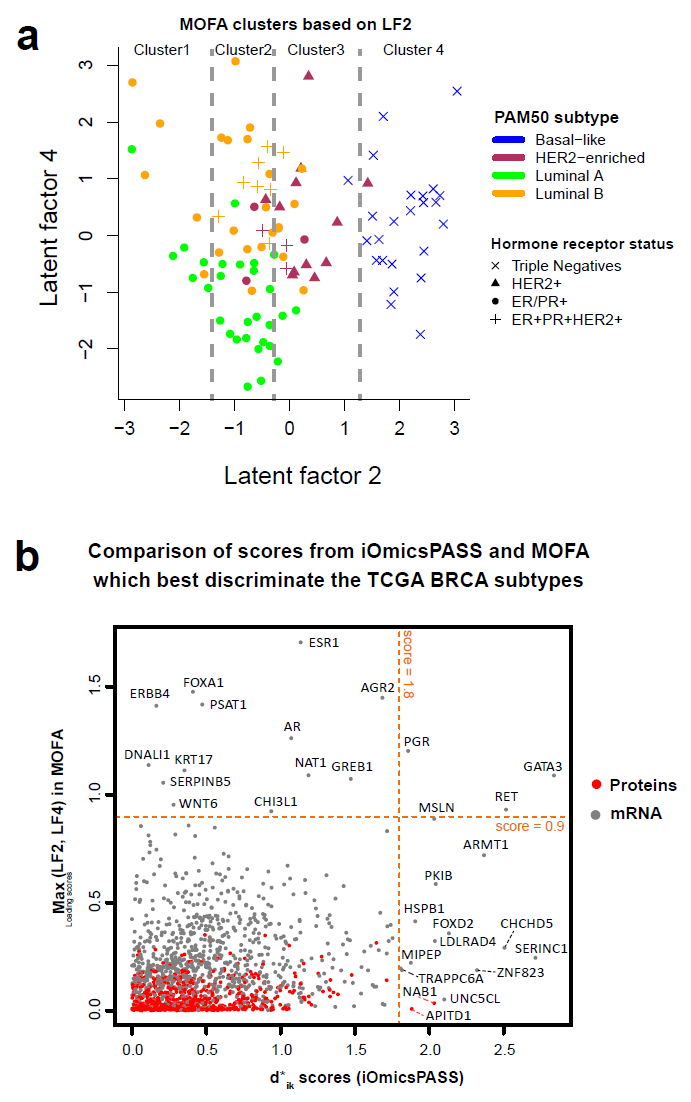


**Supplementary Figure 8**. (**a**) Subjects in TCGA BRCA projected onto the space of latent factors 2 and 4 by the MOFA analysis which appears to best differentiate the four subtypes. Latent factor 2 separates Basal-like and HER2E from the luminal subtypes and latent factor 4 separates luminal A from luminal B subtype. The distinction between subtypes is less clear than the PCA plot shown in Supplementary Figure 4A, but this is because the latter was drawn using the predictive features only. However, the two plots share the same patterns in subtype separation, where the Basal-like subtype is the most distinguished amongst all subtypes, with HER2E being a subset of Luminal subtypes (**b**) Comparison of maximal loading scores on latent factors 2 & 4 and centroid scores of iOmicsPASS. The former is a node-specific score, whereas the latter is an edge-specific score. We took the centroid score of each node for iOmicsPASS. While MOFA shows greater loading scores for mRNA molecules, iOmicsPASS shows equal contribution of the two molecules due to the biological prior imposed through the network.

**References**

1 Laurent, J. M. *et al.* Protein abundances are more conserved than mRNA abundances across diverse taxa. *Proteomics* **10**, 4209-4212, (2010).

2 Parker, J. S. *et al.* Supervised risk predictor of breast cancer based on intrinsic subtypes. *J Clin Oncol* **27**, 1160-1167, (2009).

3 Mertins, P. *et al.* Proteogenomics connects somatic mutations to signalling in breast cancer. *Nature* **534**, 55-62, (2016).
